# Investigation of *HER-2* Expression an Its Correlation with Clinicopathological Parameters and Overall Survival of Esophageal Squamous Cell Carcinoma Patients

**DOI:** 10.30699/IJP.2020.113829.2235

**Published:** 2020-07-16

**Authors:** Mitra Heidarpour, Mehran Taheri, Ali Akhavan, Parvin Goli, Amirhosein Kefayat

**Affiliations:** 1 *Department of Pathology, Faculty of Medicine, Isfahan University of Medical Sciences, Isfahan, Iran*; 2 *Department of Radiation Oncology, Faculty of Medicine, Isfahan University of Medical Sciences, Isfahan, Iran*; 3 *Department of Immunology, Faculty of Medicine, Isfahan University of Medical Sciences, Isfahan, Iran*; 4 *Department of Oncology, Cancer Prevention Research Center, Faculty of Medicine, Isfahan University of Medical Sciences, Isfahan, Iran*

**Keywords:** Esophageal cancer, Human epidermal growth factor receptor 2, Squamous cell carcinoma

## Abstract

**Background & Objective:**

Human epidermal growth factor receptor 2 (HER-2) exhibits a vast range of expression in esophageal squamous cell carcinoma (ESCC) patients as a biomarker. This paper aimed to investigate HER-2 expression and clinicopathological parameters of esophageal SCC.

**Methods:**

HER-2 expression was assessed in 102 ESCC patients by immunohistochemistry. The HER-2 staining intensity , according to the Gastric HER2 Biomarker1.0.0.1 version of the college of American pathologists (CAP) protocol for gastric and gastroesophageal junction cancers, was graded as 0 (no reactivity in any of the cancer cells’ membranes); 1+ (pale or hardly noticeable reactivity in the membrane of cancer cells’ cluster [≥ 5 neoplastic cells] regardless of the positive cancer cells’ percentage); 2+ (weak-to-moderate complete, basolateral, or lateral membranous reactivity regardless of the positive cancer cells’ percentage); and 3+ ( strong complete, basolateral, or lateral reactivity in the membrane of the cancer cell cluster regardless of the positive cancer cells’ percentage).In this regard, 3+ scored samples were considered as positive. If HER-2 expression was scored 2+, an additional fluorescence in situ hybridization (FISH) was performed. Fisher's exact test was employed for investigating the correlation of HER-2 expression status with patients’ clinicopathological characteristics (including age, gender, tumor location, stage, grade, infiltration level, venous invasion, lymphatic invasion, and tumor recurrence). Kaplan-Meier analysis was done for the patients’ survival assessments.

**Results:**

Five patients (~5%) were HER-2 positive and no significant association was observed between HER-2 expression and clinicopathological properties. In addition, HER-2 expression status exhibited no significant association with the patients’ overall survival (*P*=0.9299).

**Conclusion:**

HER-2 is not a suitable prognostic biomarker for Iranian ESCC patients.

## Introduction

Esophageal cancer has become a major public health problem in the world. According to statistical analyses, it is the sixth most common cancer worldwide and the ninth most common cause of cancer-related deaths (1). Squamous cell carcinoma (SCC) is the most frequent histological type of esophagus cancer (2). Environmental factors play a determinative role in the rising of these tumors (3). Despite intensive therapeutic interventions, including surgical resection with lymphadenectomy and different pre- and post-operation adjuvant therapies, most of the patients with the localized disease develop metastatic lesions, and the mortality rate is still high (4). Esophageal squamous cell carcinoma (ESCC) generally exhibits a poor prognosis due to its advanced stage at the time of diagnosis, and the survival rates of patients with advanced disease are not satisfactory (5).

 Human epidermal growth factor receptor 1 (HER-1), HER-2, HER-3, and HER-4 are the four homologs members of the HER family. Abnormal kinase activity in these receptors can cause ESCC tumor development, progression, and even metastasis (6). Their determinative role was demonstrated in breast cancer and led to the appearance of specific treatments based on inhibition and inactivation of these receptors (7). HER-2 proto-oncogene is a 185-kDa transmembrane glycoprotein with tyrosine-specific kinase activity (8). Its gene (HER-2/neu) locates on chromosome 17 (17q12–q21.32) (9). HER‐2 expression is detected in 22–66% of ovarian cancer patients (10). Also, its expression was detected in 26% of gastric cancers (11). 

Monoclonal antibody (mAb), with a high ability of attaching to HER-2 receptor (Herceptin®), causes anti-growth effects against HER-2 overexpressing tumors. Herceptin has attracted lots of clinicians’ attention in immunotherapy, and it is the first approved mAb for the treatment of breast cancer (12). Besides, multiple pre-clinical and clinical studies have reported that HER-2 can be immunogenic. Antibody generation and cytotoxic T lymphocytes and T helper cells’ activation against HER-2 positive cells were observed in individuals with HER-2 overexpressing tumors (13–15). Therefore, anti-HER-2 immune therapy could be utilized as a suitable therapeutic approach for cancers such as esophageal cancer. However, a vast spectrum of the HER-2 expression rate was reported for ESCC (16–18). Therefore, it is vital to determine the HER-2 expression frequency in the target population to determine the possibility of HER-2 based immunotherapy. 

To the best of our knowledge, a vast spectrum of HER-2 expression in ESCC was reported by different studies. In addition, a limited number of studies have reported the correlation of HER-2 expression with clinicopathological parameters of ESCC patients, especially in Iranian patients. This study aimed to investigate the frequency of HER-2 expression and its correlation with the clinicopathologic properties of Iranian ESCC patients.

##  Materials and Methods

**Patients and Samples**

This retrospective study was conducted at the Pathology Department of Isfahan University of Medical Sciences. This study was carried out on 102 paraffin-embedded samples, obtained from the archive of the Pathology Department, Al-Zahra Hospital. All specimens belonged to the ESCC patients, who were diagnosed and undergone surgery from January 2012 to December 2017. Patients with intact and complete clinical data (including diagnosis, age, sex, address, disease history, and pathological reports) were just included in this study. In this regard, patients who were diagnosed with other malignancies of the esophagus or died within four weeks after surgery were excluded from the study. Only tumor biopsy specimens were used in this study instead of surgically resected tumor specimens, as preoperative radiation or chemotherapy can affect the HER-2 expression status of the tumor (19, 20). All of the specimens were separately assessed by two different pathologists. Therefore, any specimen, which any inconsistency was observed between the pathologists’ ideas with its clinical data, was excluded. 

**Immunohistochemistry**

For immune staining, 3–5 mm sections of the paraffin-embedded specimens were prepared. The samples were incubated at 60ºC (40 min) and immersed in xylene (Sigma, USA) for deparaffinization. Subsequently, the samples were placed in the decreasing ethanol solutions for rehydration and deactivation of endogenous peroxidases; the samples were incubated in 0.3% hydrogen peroxide. Then, phosphate buffer saline (PBS) was used for washing the slides (Sigma, USA), and the washed slides were heated in an 830-W microwave oven for at least 15 min in 10 mmol/L sodium citrate buffer (pH 6.0) (Sigma-Aldrich, Germany) for antigen retrieval. 

The slides were incubated with mouse anti-HER-2 monoclonal antibody (Clone SP107, Master Diagnostica, Spain) overnight at 4ºC. Also, PBS was replaced by the primary antibody in some samples to have negative controls. The rabbit anti-mouse horseradish peroxidase-conjugated secondary antibody (Abcam, USA) was incubated for 40 min at room temperature. Then, the diaminobenzidine (DAB) (Sigma-Aldrich, Germany) was used as a chromogen. After each step, the slides were washed three times by PBS. 

**Immunostaining Scoring**

The immunostained sections were observed under a light microscope (Olympus, Japan) by two pathologists, who were not aware of the patients’ clinicopathological data. A double-headed microscope was used for the simultaneous assessment of the samples if there were any discrepancies between the pathologists’ reports. In this study, the HER-2 scoring was based on the Gastric HER2 Biomarker 1.0.0.1 version of the college of American pathologists (CAP) protocol for gastric and gastroesophageal junction cancers (21). The intensity of HER-2 staining, according to the CAP protocol for gastric and gastroesophageal junction cancers, was graded as follows: 0 (no reactivity in any of the cancer cells’ membranes), 1+ (pale or hardly noticeable reactivity in the membrane of cancer cells’ cluster [≥ 5 neoplastic cells] regardless of the positive cancer cells’ percentage); 2+ (weak-to-moderate complete, basolateral, or lateral membranous reactivity regardless of the positive cancer cells’ percentage); and 3+ ( strong complete, basolateral, or lateral reactivity in the membrane of the cancer cell cluster regardless of the positive cancer cells’ percentage). If HER-2 expression was scored 2+, an additional fluorescence in situ hybridization (FISH) was performed according to previous studies to confirm positivity (17).

**Statistical Analysis**

JMP software version 11.0 (SAS institute, Japan) was employed for statistical analyzes. The correlation of HER-2 expression and clinicopathological parameters was assessed by Fisher's exact test. Patients’ overall survival was calculated by using the Kaplan–Meier method and compared by the log-rank test. A P-value<0.05 was considered significant.

## Results

**HER-2 Expression in ESCC Patients**

A total of 102 ESCC patients were investigated in this study. Their clinicopathological parameters are illustrated in Table 1. The patients’ age ranged from 36 to 86 years old, and their median was 62. Further, 64% of the patients were male. More than half of the tumors were located in the middle portion of the esophagus, and the most common infiltration level (T) was T3. About 28% and 34% of the patients were positive for venous and lymph node invasion, respectively. Also, 52% of the patients exhibited tumor recurrence.

**Table 1:** Clinicopathological characteristics of the ESCC patients

Clinicopathological parameters	**Patients number (n=102)**	**Proportion** **(%)**
Age		
**Median**	62 [36-86]	-
Gender		
**Male**	65	64%
**Female**	37	36%
Tumor location		
**Upper**	17	17%
**Middle**	53	52%
**Lower**	32	31%
T		
**T1**	15	15%
**T2**	22	21%
**T3**	55	54%
**T4**	10	10%
Nodal status		
**N0**	61	60%
**N1**	26	25%
**N2**	10	10%
**N3**	5	5%
Grade		
**G1**	31	30%
**G2**	56	55%
**G3**	15	15%
Stage		
**I**	14	14%
**II**	49	48%
**III**	19	18%
**IV**	20	20%
Venous invasion		
**Negative**	74	73%
**Positive**	28	27%
Lymphatic invasion		
**Negative**	68	67%
**Positive**	34	33%
Recurrence		
**Negative**	40	39%
**Positive**	53	52%
Data not available	9	9%
ESCC = Esophageal squamous cell carcinoma.		

**Correlation of HER-2 Expression with Clinicopathological Parameters of ESCC Patients**

The patients were divided into two groups based on the HER-2 expression status (Table 2). A limited number of patients (n=5) were HER-2 positive, according to the utilized scoring method. As illustrated in Table 2, no significant (*P*>0.05) correlation was observed between HER-2 expression and clinicopathological parameters, including age (*P*=0.3525), gender (*P*=0.2524), histological grade (*P*=0.3617), infiltration level (*P*=0.5784), lymph node status (*P*=0.9927), venous (*P*=0.6929) and lymphatic invasion (*P*=0.7491), and tumor recurrence (*P*=0.4577). In this study, a few percentages of the patients were HER-2 positive, and this expression status did not exhibit a significant correlation with their clinicopathological properties.

**Table 2:** Clinicopathological parameters of the ESCC patients in the HER-2 positive and negative patients

Clinicopathological parameters	HER-2-negative [n= 97]	HER-2-positive [n=5]	P-value
Age			
**≤ 60 years**	56 (58%)	3 (60%)	0.3525
**> 60 years**	41 (42%)	2 (40%)	
Gender			
**Male**	62 (64%)	3 (60%)	0.2524
**Female**	35 (36%)	2 (40%)	
Tumor location			
**Upper**	30 (15%)	2 (40%)	0.2584
**Middle**	52 (54%)	1 (20%)	
**Lower**	15 (31%)	2 (40%)	
T			
**T1**	14 (14%)	1 (20%)	0.5784
**T2**	20 (21%)	2 (40%)	
**T3**	53 (55%)	2 (40%)	
**T4**	10 (10%)	0 (0%)	
Nodal status			
**N0**	58 (60%)	3 (60%)	0.9927
**N1-3**	39 (40%)	2 (40%)	
Grade			
**G1**	30 (31%)	1 (20%)	0.3617
**G2**	54 (56%)	2 (40%)	
**G3**	13 (13%)	2 (40%)	
Stage			
**I**	14 (14%)	0 (0%)	0.6688
**II**	46 (47%)	3 (60%)	
**III**	18 (19%)	1 (20%)	
**IV**	19 (20%)	1 (20%)	
Venous invasion			
**Negative**	70 (72%)	4 (80%)	0.6929
**Positive**	27 (28%)	1 (20%)	
Lymphatic invasion			
**Negative**	65 (67%)	3 (60%)	0.7491
**Positive**	32 (33%)	2 (40%)	
Recurrence			
**Negative**	37 (38%)	3 (60%)	0.4577
**Positive**	51 (53%)	2 (40%)	
Data not available	9 (9%)	0 (0%)	

**Predictive Value of HER-2 Expression Status for ESCC Patients’ Survival**

**Fig. 1:**
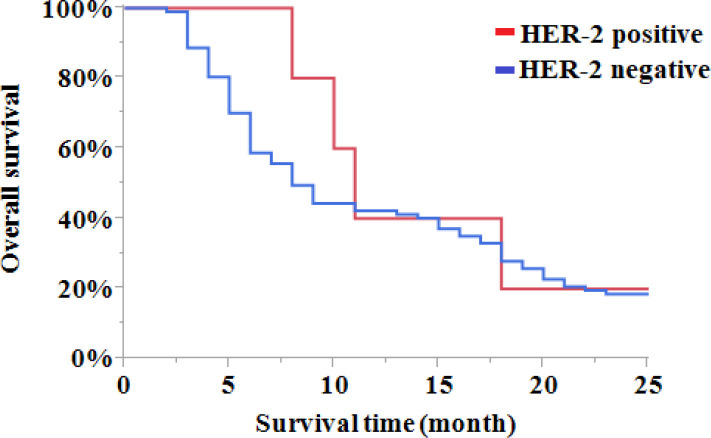
The overall survival of patients in the HER-2 positive and negative groups within 24 months after diagnosis. (Log-Rank=0.0077, *P*=0.9299)

The correlation of HER-2 expression status and the patients’ survival were investigated within 24 months after diagnosis date by searching patients’ disease history. KaplanMeier survival analysis with a log-rank test was used for this purpose. As Figure 1 illustrates, the overall survival rates were up to 20% (1/5) and 30% (79/97) for the HER-2 positive and negative patients, respectively. Therefore, HER-2 expression status was not significantly correlated with ESCC patients’ overall survival (*P*=0.9299).

## Discussion

The HER family plays a key role in epithelial cell growth, proliferation, and differentiation. Therefore, it would be a good choice for targeted therapy. HERs are transmembrane proteins with an extracellular domain for binding to the ligands and intracellular tyrosine kinase domain (22). A ligand binding to a single receptor induces conformational changes and dimerization, which activates the kinase activity of the intracellular domain and downstream signaling cascade. Among all four HER family proteins, HER-2 has the strongest activity (23). 

Overamplification of this gene significantly affects tumors’ development. HER-2 amplification has been described in tissue samples from different malignancies, such as breast, gastric, and pancreatic cancers (24–27). Further investigation demonstrated the correlation of HER-2 overexpression and poor prognosis in ovarian and breast cancers (28,29). Amplification of the HER-2 antigen has been detected in 15–30% of invasive breast cancers (30). The wide range of expression reflects that HER-2 has a vast spectrum of expression rates in different populations. The frequency of HER-2 overexpression in ESCC has been reported to vary from 0% up to about 65%, according to immunohistochemistry (IHC) investigations (31–35). 

In a report describing the HER-2 status in ESCC, HER-2 overexpression was correlated with extramural invasion and poor response to neoadjuvant chemotherapy (32). However, there is an apparent controversy regarding the expression of HER-2 receptor in esophageal carcinoma. Gibault* et al. *(36) and Reichelt* et al. *(37) observed positive expression of the HER-2 receptor in 2.8% and 7% of the involved ESCC patients in their studies, respectively. Nonetheless, some studies reported positive expression of the HER-2 marker in more than 50% of their included patients’ tumors (31). Multiple reasons can be mentioned for explaining these significant differences, including patient selection methods, immunostaining procedures, and scoring protocols. 

Wu* et al. *(38) observed the HER-2 overexpression in 14.1% of the ESCC patients. Also, Yoon* et al. *(39) and Zhan* et al. *(33) reported about 17% and 10% of the HER-2 overexpression in the esophageal adenocarcinomas and ESCC tumors, respectively. In a study by Mimura *et al.*, only 9 cases of 66 primary ESCC tumors were HER-2 positive. Three of these cases scored 3+, which exhibited HER-2 overexpression in the metastatic lymph nodes. The other six cases were 2+ score in the primary tumors; four cases had metastases, and only two out of four lymph node metastases retained 2+ HER-2 expression. The other two cases exhibited negative HER-2 staining (32). 

Different studies have mentioned various conclusions regarding the correlation of HER-2 expression and clinicopathological parameters of ESCC patients. Wu* et al. *reported no significant correlation between the ESCC patients’ tumor HER-2 overexpression and their clinicopathological characteristics (38). On the other hand, Zhan* et al. *observed significant correlations between HER-2 expression status of tumors and their differentiation level and stage (33). According to a study at the Mayo Clinic, esophageal adenocarcinoma patients with positive HER-2 expression exhibited lower tumor aggressiveness and higher survival time (39). Another study identified low HER-2 amplification in ESCC patients and its correlation with tumor infiltration depth and vascular and lymph node metastases (40). However, Barros Silva reported that HER-2 amplification does not have a significant correlation with the gastric cancer patients’ age, gender, staging, or lymph node metastasis (41). 

According to our study, which was carried out on 102 ESCC patients, a limited number of patients were HER-2-positive, which comprised 5% of the studied cases. However, there was no correlation between the HER-2 expression and the clinicopathological features. About 28% and 34% of the patients were positive for venous and lymph node invasion, respectively. Also, 52% of the patients exhibited tumor recurrence. However, no difference was observed between HER-2 positive and negative patients in these parameters.

## Conclusion

The analysis of HER-2 expression exhibited a significantly lower number of HER-2 positive cases in the Iranian ESCC patients compared with previous studies in other populations. No correlation was observed between HER-2 status and age, gender, tumor location, infiltration level, stage, grade, lymph node status, venous, lymphatic invasion, and overall survival of the ESCC patients. Taking together, HER-2 is not an efficient prognostic biomarker and potential therapeutic target for Iranian ESCC patients. To confirm these findings, more comprehensive studies are needed.
